# Spectral-DETR: Learnable Frequency Decomposition with Adaptive Contrastive Regularization for Robust Underground Mine Detection

**DOI:** 10.3390/jimaging12090401

**Published:** 2026-08-26

**Authors:** Yuexin Song, Lukang Dai, Xinqi Xu, Jun Yang

**Affiliations:** 1 School of Artificial Intelligence, Haidian Campus, China University of Mining and Technology (Beijing), Beijing 100083, China; 2310310319@student.cumtb.edu.cn (Y.S.); 2210570718@student.cumtb.edu.cn (L.D.); 2410403121@student.cumtb.edu.cn (X.X.); 2 Big Data and Internet of Things Research Center, China University of Mining and Technology (Beijing), Beijing 100083, China; 3 Key Laboratory of Intelligent Mining and Robotics for Coal Mines, Ministry of Emergency Management, Beijing 100083, China

**Keywords:** low-light object detection, frequency-domain learning, contrastive learning, uncertainty calibration, DETR, underground mine perception

## Abstract

Underground mine object detection is challenged by low illumination, blur, dust scattering, and repetitive tunnel clutter, which jointly corrupt backbone features, entangle DETR queries, and weaken localization for small objects. Existing enhancement-based and detector-internal methods do not explicitly propagate degradation reliability across features, decoder queries, and box refinement. We propose Spectral-DETR, a detector-internal reliability framework built on RF-DETR. Its central design is a cross-stage reliability pathway that connects Degradation-Aware Frequency Decomposition (DAFD), Degradation-Adaptive Query Contrastive Denoising (DQCD), and Salience-Calibrated Uncertainty with Learned Uncertainty Estimation (SCU+LUE). On Mine-Objects (14 classes, 3081 images), Spectral-DETR achieves an average precision of 0.917 at an intersection-over-union threshold of 0.5 and 0.493 when averaged over thresholds from 0.5 to 0.95, exceeding YOLOv9m by 1.6 and 0.8 percentage points, respectively, under the dataset-specific evaluation protocol. In controlled RF-DETR validation, the three reliability stages improve these two measures from 0.883 to 0.913 and from 0.472 to 0.486, respectively. Spectral-DETR obtains corresponding values of 0.848 and 0.571 on ExDark and 0.973 and 0.495 on ScienceDB. DQCD and SCU remain training-only losses with no inference cost.

## 1. Introduction

Reliable object detection is safety-critical in underground mines, where missed detections of personnel, warning signs, and electrical equipment directly affect hazard-warning systems and personnel safety. Underground imagery is captured under coupled degradations (low illumination, motion blur, dust scattering, and repetitive tunnel clutter) that suppress local contrast, weaken object boundaries, and obscure foreground structure. Detectors developed under clean imaging assumptions suffer a substantial robustness gap when deployed underground [1,2,3].

This gap is particularly severe for DETR-style end-to-end detectors [4]. In degraded scenes, performance loss is not caused by an isolated component; instead, degradation progressively breaks the detector’s internal reliability chain at three coupled points: (i) **feature corruption**: backbone features are degraded by frequency-specific effects [1,2] (low-frequency illumination attenuation, mid-frequency texture erosion from blur, high-frequency noise from dust) before they feed decoder query formation; (ii) **representation entanglement**: matched foreground queries and unmatched background queries become tangled in the decoder embedding space, especially when hard negative locations are visually similar to degraded foreground objects; and (iii) **localization under-optimization**: after successful query matching, the most difficult small/medium targets still receive insufficient refinement because standard L1 and GIoU losses distribute gradient effort uniformly across all positives [4,5].

Existing approaches address related difficulties through low-light enhancement and degraded-image learning [2,6], detector architecture redesign [7,8], uncertainty-aware regression [9,10], and frequency-inspired learning [11,12]. These efforts are valuable but leave three limitations in our setting. First, enhancement modules typically operate outside the detector and may introduce artifacts that are not aligned with the detection objective. Second, uncertainty modeling in DETR-style detectors remains under-explored [4,13,14]; most formulations target anchor-based CNN detectors or treat uncertainty as a passive quality score [15] rather than as a localization-weighting signal. Third, frequency-domain learning usually focuses on feature transformation alone [11,12] and does not connect learned frequency reliability to downstream query regularization. What is still missing is a detector-internal framework that treats degraded detection as a reliability propagation problem across features, queries, and localization.

To address this gap, we propose **Spectral-DETR**, a detector-internal reliability framework built on RF-DETR [16]. Rather than adding a separate image-restoration front-end, Spectral-DETR implements one reliability pathway across three points where degradation affects the detection pipeline:

- **Feature reliability**: DAFD performs FFT-based decomposition into three learnable Gaussian bands, applies per-band scene-adaptive FiLM and gating, reconstructs each band by IFFT, and fuses the spatial bands through a cross-band convolution with a scaled residual. The band geometry, FiLM encoders, gates, and fusion layer are trained end-to-end by detection loss gradients.
- **Query reliability**: DQCD applies supervised contrastive learning to final decoder query embeddings. Its InfoNCE temperature is modulated by DAFD gate statistics, so images with stronger frequency suppression receive stronger query separation. DQCD requires no paired views, teacher model, or auxiliary network and is used only during training.
- **Localization reliability**: SCU+LUE predicts coordinate-level log-variances, calibrates them using geometric salience, and precision-weights L1 regression for small/medium objects while retaining standard GIoU supervision. This turns uncertainty estimation into an explicit localization-refinement signal rather than only a diagnostic output.

The contributions are:

- We formulate degraded underground detection as detector-internal reliability propagation and make the cross-stage pathway explicit: DAFD gate statistics condition DQCD temperature, while SCU-calibrated LUE weights localization supervision.
- We provide a task-driven multi-band frequency implementation (DAFD) and a decoder-query contrastive objective (DQCD) whose individual operators are established ideas adapted to this coupled detector setting.
- We provide SCU+LUE as a geometric calibration and precision-weighting mechanism for small/medium-object localization, and evaluate the individual stages, pairwise combinations, and coupling controls under a common RF-DETR protocol.

## 2. Related Work

### 2.1. Transformer-Based Detection and Degraded-Scene Robustness

Before transformer-based detectors, two-stage and one-stage CNN detectors established the main accuracy-efficiency trade-offs in object detection, including Fast/Faster R-CNN [17,18], SSD [19], RetinaNet [20], FCOS [21], EfficientDet [22], and YOLO-family detectors [23,24,25,26,27]. DETR [4] and its variants, including Deformable DETR [13], DAB-DETR [14], DN-DETR [28], DINO [29], and RT-DETR [30,31], have improved convergence and efficiency through deformable attention, dynamic anchors, query denoising, and real-time architectures. Co-DETR [32] and H-DETR [33] address training efficiency through hybrid label assignment. RF-DETR [16] provides a strong DINOv2-based baseline [34] with multi-scale projection and two-stage query selection. The Vision Transformer [35] and its hierarchical variants [36] have also inspired detector backbones, while Feature Pyramid Networks [37] remain foundational for multi-scale feature extraction.

For robustness under degradation, low-light enhancement [2,6,38,39,40] and frequency-domain modeling [11,12] have made progress. Attention mechanisms such as SE, CBAM, and FcaNet also show that channel and frequency statistics can improve visual representations [41,42,43]. More recent detector-specific studies narrow the novelty space further. MPE-DETR uses multiscale pyramid enhancement for low-light detection [44]; MDFD2-DETR decomposes and de-redundifies multi-domain features for complex road scenes [45]; and frequency-enhanced Transformers have been developed for nighttime and low-light detection [46,47]. Spectral-DETR therefore does not claim that frequency processing inside a detector is itself novel. Its distinction is the combination of overlapping learnable Gaussian bands, scene-conditioned per-band gates on intermediate encoder taps, and an explicit downstream interface in which the gate statistic conditions query-level contrastive temperature. SCU+LUE then extends reliability weighting to localization. This cross-stage information path, rather than any individual FFT or uncertainty operator, defines the contribution relative to contemporary work.

Beyond single-modal detection, multimodal object detection has been explored for degraded-scene perception by fusing complementary modalities such as RGB-D, RGB-T, and RGB-IR. CCAFNet [48] and IRFR-Net [49] develop crossflow and recursive feature-reshaping fusion for RGB-D salient object detection; ECFFNet [50], LSNet [51], and WaveNet [52] combine RGB with thermal infrared through consistent feature fusion, lightweight spatial boosting, and wavelet-based knowledge distillation for RGB-T salient object detection; LSINet [53] and RCNet [54] address rail and road surface-defect detection with lightweight scope integration and dual-network resonance collaboration; Priors Meet Hindsight [55] adopts asymmetric collaborative evolution for RGB-D road defect detection; and Turbidity–Similarity Decoupling [56] and SAM-CLNet [57] target underwater salient object detection through feature-consistent mutual learning and prompt-free SAM enhancement. These works demonstrate that complementary modalities can mitigate single-sensor degradation. However, our setting differs in two respects: (i) underground mine platforms typically carry only monocular RGB cameras due to cost, explosion-proofing, and payload constraints, making multimodal approaches inapplicable; and (ii) Spectral-DETR addresses single-modal reliability propagation inside the detector, specifically for DETR-style architectures. Multimodal fusion is a complementary direction that could be integrated with our reliability framework in future work, but it is not the focus of the present study.

### 2.2. Contrastive Learning in Object Detection

Contrastive learning has improved representation quality in visual representation learning and detection [58,59]. DetCo [59], for example, applies instance-level contrastive pretraining, while widely used contrastive objectives commonly retain a fixed global temperature [60]. DQCD instead operates on decoder query embeddings and uses an image-conditioned temperature derived from DAFD gate statistics. It therefore regularizes the representations used directly for classification and localization, without requiring a teacher model, paired views, or an auxiliary inference network. We do not present query-level contrastive learning itself as a new primitive; the contribution is its detector-specific construction and its explicit coupling to the upstream frequency-reliability signal.

### 2.3. Uncertainty Estimation for Localization

Aleatoric uncertainty modeling [9] motivated detection-specific formulations including KL-based box learning [10] and IoU-aware quality estimation [15,61]. These methods show that localization quality is spatially non-uniform. However, most DETR-style detectors still use deterministic L1+GIoU [4,5] supervision that weights all positives uniformly. SCU+LUE adapts this established uncertainty-regression idea to the present DETR pipeline by combining coordinate-level log-variance prediction, batch-centered precision weights, and a geometric-salience calibration target. The method is intended to improve relative localization weighting; uncertainty correlation is treated as a supporting diagnostic rather than conclusive calibration evidence.

## 3. Proposed Method

### 3.1. Overview and Reliability Propagation Framework

We formulate degraded underground detection as detector-internal reliability propagation through three coupled stages. Let $x\in R^{B\times 3\times H\times W}$ denote a batch of input images, where *B*, *H*, and *W* are the batch size, image height, and image width, respectively. The DINOv2 backbone exposes four intermediate encoder taps $\left\{f_{1},\ldots ,f_{4}\right\}$, and DAFD processes these taps before the multi-scale projector. The resulting projected features feed the decoder and the two training-time reliability objectives:

1. **DAFD (Feature Reliability)**: Learnable three-band frequency decomposition with scene-adaptive gating, applied before multi-scale projection. Suppresses frequency-specific degradation while preserving task-relevant structure.
2. **DQCD (Representation Reliability)**: Adaptive contrastive regularization over decoder query embeddings. DAFD’s gate statistics modulate the temperature, providing stronger foreground-background separation on more degraded images.
3. **SCU + LUE (Localization Reliability)**: Coordinate-level log-variances calibrated by geometric salience, precision-weighting the L1 regression loss for small/medium objects.

**Figure 1:**
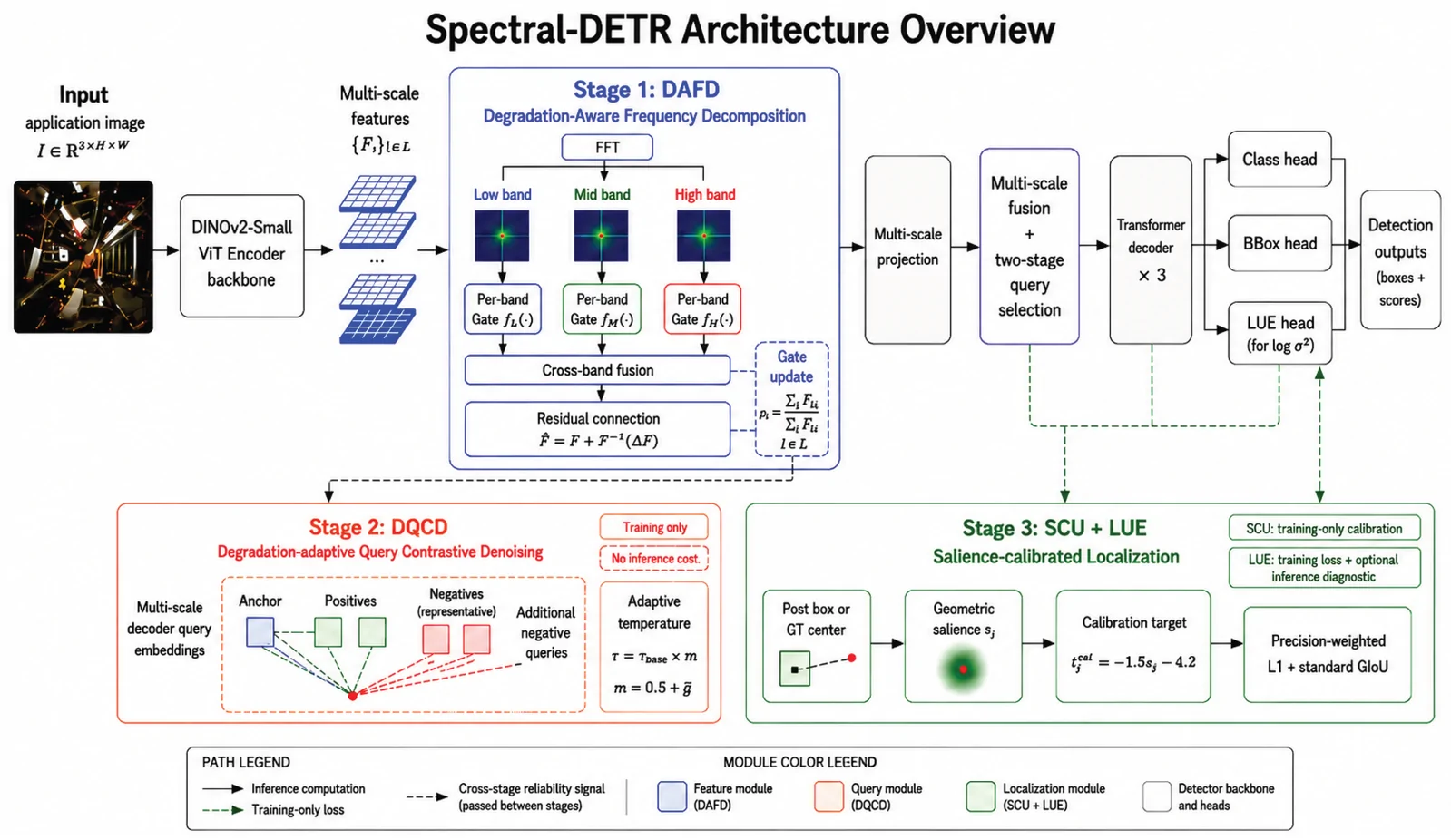
Overall architecture of Spectral-DETR. DAFD improves frequency-aware features, DQCD regularizes decoder queries with a DAFD-modulated temperature, and SCU+LUE calibrates uncertainty for precision-weighted localization. Solid arrows denote inference computation; green and black dashed arrows denote training-only objectives and cross-stage reliability signals, respectively. Module colors identify DAFD (blue), DQCD (orange), SCU+LUE (green), and detector components (gray). Only representative negative queries are shown.

**Figure 2:**
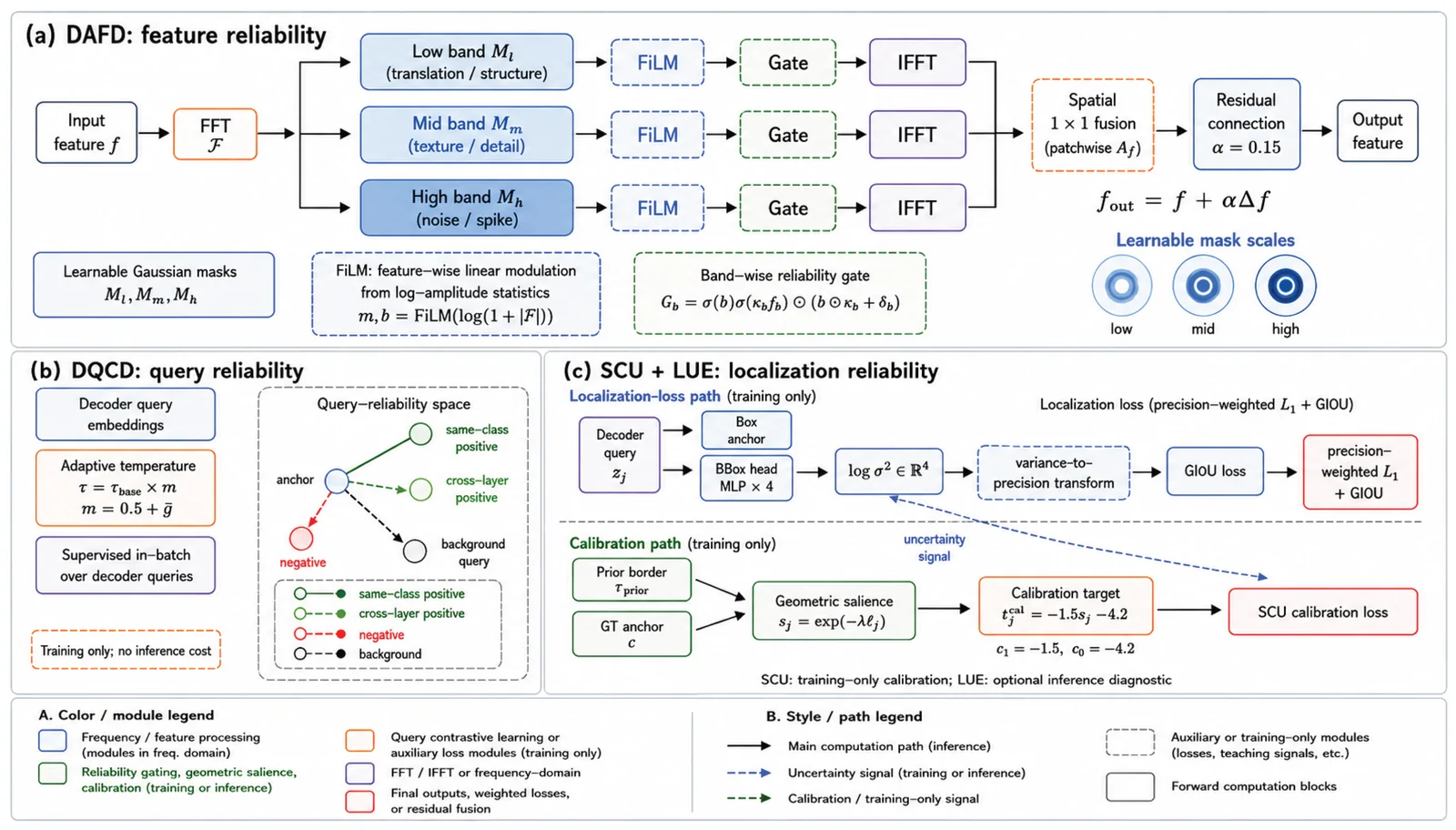
Module design of Spectral-DETR. (**a**) DAFD applies learnable Gaussian bands, FiLM, reliability gates, IFFT reconstruction, and cross-band fusion. (**b**) DQCD applies DAFD-conditioned InfoNCE regularization to decoder queries during training [62]. (**c**) SCU+LUE predicts coordinate-level log-variances, uses them to precision-weight the L1 term, and retains standard GIoU. Localization and calibration paths are training-only; LUE outputs may optionally be retained for inference diagnostics. Dashed outlines denote auxiliary or training-only components, and solid outlines denote forward-computation blocks.

Figure 1 illustrates the overall architecture. The modules are complementary: DAFD improves feature quality entering the decoder, DQCD improves the separability of resulting query representations, and SCU+LUE improves the precision of final box predictions. Critically, DAFD exposes per-band gate statistics that DQCD uses for temperature modulation, forming a direct information pathway from the frequency domain to contrastive learning. Figure 2 summarizes the three module designs.

### 3.2. DAFD: Degradation-Aware Frequency Decomposition

The first stage addresses feature-level corruption. In underground imagery, frequency-specific degradation affects different bands: low illumination attenuates low-frequency structure, motion blur erodes mid-frequency texture, and dust scattering injects high-frequency noise. A fixed filter cannot adapt to the varying mixture of these effects. DAFD is designed as a fully learnable frequency-domain module (Figure 2a).

#### 3.2.1. Multi-Band Frequency Decomposition

Given a backbone feature map $f\in R^{B\times C\times H_{f}\times W_{f}}$, where *C*, $H_{f}$, and $W_{f}$ denote channel count and feature-map dimensions, DAFD applies a two-dimensional real-valued fast Fourier transform (2D Real FFT). Because the input is real, conjugate symmetry permits retaining only $\lfloor W_{f}/2\rfloor +1$ coefficients along the last frequency axis:

(1) $$F=F_{\mathrm{rfft}}\left(f\right)\in C^{B\times C\times H_{f}\times W_{\mathrm{fft}}},$$

where $W_{\mathrm{fft}}=\left\lfloor W_{f}/2\right\rfloor +1$. Indices $u\in \{0,\ldots ,H_{f}-1\}$ and $v\in \{0,\ldots ,W_{\mathrm{fft}}-1\}$ identify frequency coordinates; $\xi_{u}$ and $\eta_{v}$ are their normalized signed frequencies produced by fftfreq and rfftfreq, respectively. The radial frequency is

(2) $$r\left(u,v\right)=\sqrt{\xi_{u}^{2}+\eta_{v}^{2}}.$$

Three frequency bands are defined by learnable Gaussian soft masks with raw center parameters $c=[c_{0},c_{1},c_{2}]$ and width parameters $w=[w_{0},w_{1},w_{2}]$. After sigmoid rescaling:

(3) $${\tilde{c}}_{k}=0.6\cdot \sigma \left(c_{k}\right)+0.05\in \left[0.05,0.65\right],\;{\tilde{w}}_{k}=0.3\cdot \sigma \left(w_{k}\right)+0.05\in \left[0.05,0.35\right],$$

(4) $$M_{k}\left(u,v\right)=\exp\;\left(-\frac{1}{2}{\left(\frac{r\left(u,v\right)-{\tilde{c}}_{k}}{{\tilde{w}}_{k}+\epsilon }\right)}^{2}\right),\;k\in \left\{0,1,2\right\},$$

where σ(·) is the sigmoid function. The Gaussian masks are intentionally not normalized across bands. This allows overlapping soft responses and lets the learned gates truly suppress or preserve spectral energy, instead of forcing all frequency coordinates to be partitioned with unit total mass. The raw band parameters are initialized in increasing order with centers [0.10,0.35,0.60] and widths [0.12,0.18,0.25], providing a physically grounded low/mid/high-frequency prior before end-to-end task-driven reallocation.

#### 3.2.2. Per-Band Gating with Scene-Adaptive FiLM

For each band *k*, frequency components are extracted via $F_{k}=F⊙M_{k}$, where ⊙ denotes element-wise multiplication with broadcasting over batch and channel dimensions. Feature-wise Linear Modulation (FiLM) applies a learned channel-wise affine transformation. Here, a scene encoder (AdaptiveAvgPool2d → Linear → ReLU → Linear, reduction ratio 4) predicts the FiLM scale $\gamma_{k}$ and shift $\beta_{k}$ from the log-amplitude spectrum $A_{k}=\log(1+|F_{k}\left|\right)$:

(5) $$\left(\gamma_{k},\beta_{k}\right)={\mathrm{SceneEncoder}}_{k}\left(A_{k}\right).$$

A per-band gating network (Conv_1×1_ → ReLU → Conv_1×1_) predicts raw gates conditioned by FiLM:

(6) $$G_{k}=\sigma \left({\mathrm{Gate}}_{k}\left(A_{k}\right)⊙\gamma_{k}+\beta_{k}\right),$$

where σ is the sigmoid function. The final gate convolution is initialized with zero weights and bias logit(0.8), so every gate initially preserves approximately 80% of its band response while retaining non-saturated gradients. The gated band is ${\tilde{F}}_{k}=F_{k}⊙G_{k}$.

#### 3.2.3. Cross-Band Fusion and Reconstruction

Each gated frequency band is independently reconstructed to the spatial domain via inverse FFT, then fused in the spatial domain to avoid complex-valued convolutions:

(7) $${\hat{f}}_{k}=F_{\mathrm{irfft}}\left({\tilde{F}}_{k}\right)\in R^{B\times C\times H_{f}\times W_{f}},$$

(8) $$f_{\mathrm{fused}}={\mathrm{Conv}}_{1\times 1}\left([{\hat{f}}_{0};{\hat{f}}_{1};{\hat{f}}_{2}]\right)\in R^{B\times C\times H_{f}\times W_{f}}.$$

The fused spatial-domain representation is added through a scaled residual connection:

(9) $$f_{\mathrm{out}}=f+\alpha f_{\mathrm{fused}},$$

where α=0.15. The cross-band fusion is zero-initialized, so DAFD starts as identity and gradually learns to filter. Performing IFFT *before* fusion (rather than fusing in frequency domain) avoids complex-valued convolutions while preserving the band-specific information through the channel-wise concatenation. A sparsity regularizer prevents gate collapse:

(10) $$L_{\mathrm{sparsity}}=\frac{1}{3}\sum_{k=0}^{2}{\left(E[G_{k}]-0.8\right)}^{2},$$

where $E[G_{k}]$ denotes the mean over the batch, channel, and two frequency dimensions. This term is weighted by $10^{-5}$ in the total objective.

#### 3.2.4. DAFD Design Rationale

Three properties distinguish DAFD: (1) **Task-driven band learning**: band boundaries are not fixed heuristics; they are optimized by detection loss gradients, enabling task-specific frequency allocation. (2) **Scene-adaptive per-band modulation**: each band receives independent FiLM conditioning based on its own spectral statistics, allowing differential treatment (e.g., boosting low frequencies under darkness while suppressing high frequencies under dust). (3) **End-to-end single-stage training**: no pretraining, auxiliary reconstruction loss, or paired views required. Per-band gate statistics $\left(E\left[G_{k}\right]\right)$ are exposed downstream for DQCD temperature modulation.

### 3.3. DQCD: Degradation-Adaptive Query Contrastive Denoising

The second stage addresses query representation entanglement. In degraded scenes, hard negative background queries (locations visually similar to degraded foreground objects) crowd the embedding space. Standard contrastive learning uses fixed temperature, ignoring per-image variation in degradation severity.

DQCD applies a supervised InfoNCE objective over final-layer decoder query embeddings (Figure 2b). Crucially, temperature is adaptively modulated by DAFD’s gate statistics. We retain the term “denoising” because DQCD regularizes the query embedding space by pushing ambiguous background negatives away from foreground anchors, a form of representation denoising analogous to its use in self-supervised learning [58].

#### 3.3.1. DAFD-Modulated Temperature

Let $\left\{G_{0},G_{1},G_{2}\right\}$ be DAFD’s per-band gate maps from encoder tap 0, the configured gate-source tap in the final model. The average gate activation across all bands and all non-batch dimensions is computed independently for each image:

(11) $$\bar{g}=\frac{1}{3}\sum_{k=0}^{2}E\left[G_{k}\right].$$

$\bar{g}$ is a learned frequency-preservation statistic, not a calibrated ground-truth degradation score. Larger values indicate that more band response passes through the learned gates. The DAFD-modulated temperature is

(12) $$\tau =\tau_{\mathrm{base}}\cdot m,\;m=\left\{\begin{array}{cc} 0.5+1.0\cdot \bar{g}, & \mathrm{if}\;\mathrm{DAFD}\;\mathrm{is}\;\mathrm{active},\\ 1.0, & \mathrm{otherwise}. \end{array}\right.$$

where $\tau_{\mathrm{base}}=0.15$. At the initialization value $\bar{g}\approx 0.8$, τ≈0.195; smaller preservation values produce lower temperatures and sharper contrastive separation. When DAFD is disabled, the modulation falls back to m=1, recovering fixed-temperature InfoNCE. Fixed, image-aligned adaptive, shuffled, and random-gate controls are included in the revision experiment protocol to determine whether the cross-stage signal contributes beyond the two modules themselves.

#### 3.3.2. Contrastive Objective

Let $h_{i}\in R^{C}$ denote the L2-normalized final decoder layer embedding of matched query *i*. Positives $P_{i}$ include:

- **Cross-layer positives**: the same query’s embeddings from preceding decoder layers near the final layer, providing always-available positives that avoid the “no same-class positives” failure mode common in detection.
- **Same-class positives**: other matched queries of the same class in the batch.

Negatives $N_{i}$ are the unmatched background queries from the same image as anchor *i*. For each anchor, hard negatives are ranked by $h_{i}^{⊤}h_{n}+0.25a_{n}$, where $h_{i}^{⊤}h_{n}$ denotes the vector inner product and $a_{n}=1-\left|p_{n,1}-p_{n,2}\right|$ is classification ambiguity computed from the top two sigmoid class probabilities. The centered dot used elsewhere denotes ordinary scalar multiplication. The top K=128 negatives are retained; if fewer unmatched queries are available, all are used.

The InfoNCE loss for anchor *i*:

(13) $$L_{\mathrm{DQCD}}^{\left(i\right)}=-\log\frac{\sum_{p\in P_{i}}\exp\left(h_{i}^{⊤}h_{p}/\tau \right)}{\sum_{p\in P_{i}}\exp\left(h_{i}^{⊤}h_{p}/\tau \right)+\sum_{n\in N_{K}}\exp\left(h_{i}^{⊤}h_{n}/\tau \right)}.$$

The unweighted DQCD loss averages over matched anchors:

(14) $$L_{\mathrm{DQCD}}=\frac{1}{\left|A\right|}\sum_{i\in A}L_{\mathrm{DQCD}}^{\left(i\right)},$$

where A is the set of matched anchors. The final loss coefficient is $\lambda_{\mathrm{DQCD}}=0.08$. Cross-layer positives ensure that an anchor remains valid even when no other same-class match exists in the mini-batch.

DQCD is zero before epoch 8 and is then linearly warmed to its full coefficient over five epochs. It is a training-only objective with zero inference cost.

### 3.4. SCU + LUE: Salience-Calibrated Precision-Weighted Localization

The third stage addresses localization under-optimization. Standard L1 regression weights all positives equally, but degraded small/medium targets require more refinement pressure. We design a two-component system: LUE predicts coordinate-level log-variances for precision-weighted regression, and SCU provides explicit calibration targets (Figure 2c).

#### 3.4.1. LUE: Learned Uncertainty Estimation

An MLP head (3-layer, hidden_dim → hidden_dim → 4, identical architecture to the box head) predicts 4-dimensional log-variances $\log\sigma^{2}\in R^{4}$ per query:

(15) $$\log\sigma^{2}={\mathrm{MLP}}_{\mathrm{var}}\left(h_{\mathrm{dec}}\right).$$

The head is initialized with zero weights and bias −5.0, giving initial variance exp(−5.0)≈0.0067. This prevents the “uncertainty trap” where under-confident predictions receive no gradient early in training.

**Precision-Weighted L1 Regression.** During training, log-variances are batch-centered for discriminability:

(16) $$\log{\tilde{\sigma }}^{2}=\log\sigma^{2}-\bar{\log\sigma^{2}},$$

then clamped and exponentiated to precision:

(17) $$p=\exp\left(-\mathrm{clamp}\left(\log{\tilde{\sigma }}^{2},-1.5,1.1\right)\right)\in \left[0.33,4.48\right].$$

The clamp bounds produce a 13.6× precision range, sufficient for meaningful differentiation without gradient instability. Precision is mean-normalized:

(18) $$\hat{p}=\frac{p}{E[p]+\epsilon }.$$

An adaptive area threshold determines which objects receive precision weighting:

(19) $$\tau_{\mathrm{area}}=\mathrm{clamp}\left(3.0\cdot \mathrm{median}({\left\{w_{j}h_{j}\right\}}_{j=1}^{N_{\mathrm{pos}}}),\;0.02,\;0.15\right).$$

This threshold is computed from the median normalized area of the matched positive targets in the current batch and therefore adapts batch-wise. For example, when the batch median area is approximately 0.003, the unclamped value is approximately 0.009 and is clipped to the lower bound, yielding ($\tau_{\mathrm{area}}=0.02$); for a median area of approximately 0.03, ($\tau_{\mathrm{area}}\approx 0.09$); and for approximately 0.05, ($\tau_{\mathrm{area}}$) reaches the upper bound of 0.15. The small/medium mask is defined as $m_{j}=⊮\left[w_{j}h_{j}<\tau_{\mathrm{area}}\right]$.

The precision-weighted L1 loss is computed for small/medium objects, with a warmup-controlled blend between precision-weighted and plain L1:

(20) $$\begin{array}{cc} L_{\mathrm{bbox}}^{w} & =\frac{1}{4}\sum_{d=1}^{4}\left[m_{j}\cdot {\hat{p}}_{j,d}\cdot |b_{j,d}^{\mathrm{pred}}-b_{j,d}^{\mathrm{gt}}|+\left(1-m_{j}\right)\cdot \left|b_{j,d}^{\mathrm{pred}}-b_{j,d}^{\mathrm{gt}}\right|\right], \end{array}$$

(21) $$\begin{array}{cc} L_{\mathrm{bbox}}^{0} & =\frac{1}{4}\sum_{d=1}^{4}\left|b_{j,d}^{\mathrm{pred}}-b_{j,d}^{\mathrm{gt}}\right|, \end{array}$$

(22) $$\begin{array}{cc} L_{\mathrm{bbox}} & =\frac{1}{N_{\mathrm{pos}}}\sum_{j=1}^{N_{\mathrm{pos}}}\left[\rho \cdot L_{\mathrm{bbox}}^{w}+\left(1-\rho \right)\cdot L_{\mathrm{bbox}}^{0}\right], \end{array}$$

where $\rho =\min\left(1,\mathrm{epoch}/T_{\mathrm{warmup}}\right)\in \left[0,1\right]$ is the LUE warmup ratio and $T_{\mathrm{warmup}}=15$. During warmup, ρ ramps from 0 to 1, smoothly transitioning from plain L1 to precision-weighted L1. This blending ensures the box head stabilizes before precision weighting takes effect. The standard GIoU loss is retained unchanged.

**Uncertainty Calibration Loss.** Log-variances are supervised against a bounded log-error target. For each coordinate, the detached L1 error is clamped after the logarithm to [−7,0], and Smooth L1 with transition parameter δ=0.5 is used rather than an unbounded squared error:

(23) $$q_{j,d}=\mathrm{clamp}\;\left(\log\;\left(\mathrm{sg}(|b_{j,d}^{\mathrm{pred}}-b_{j,d}^{\mathrm{gt}}|)+\epsilon \right),-7,0\right),$$

(24) $$L_{\mathrm{unc}}=\frac{\rho }{N_{\mathrm{pos}}}\sum_{j=1}^{N_{\mathrm{pos}}}m_{j}\cdot \frac{1}{4}\sum_{d=1}^{4}{\mathrm{SmoothL1}}_{\delta =0.5}\left(\log\sigma_{j,d}^{2},q_{j,d}\right),$$

where sg(·) denotes stop-gradient and ϵ is a numerical floor. The lower clamp avoids unreachable targets as coordinate errors approach zero. The same warmup ratio ρ gates this loss, and the standard GIoU loss remains unchanged in the final configuration.

#### 3.4.2. SCU: Salience-Calibrated Uncertainty

LUE learns variances passively from regression errors. However, uncertainty should also reflect geometric difficulty: a prediction far from the ground-truth center should carry higher uncertainty than a perfectly centered one, even at similar L1 error. SCU provides this prior.

For matched query *j*, geometric salience is:

(25) $$s_{j}=\exp\;\left(-\frac{∥c_{j}^{\mathrm{pred}}-c_{j}^{\mathrm{gt}}{∥}_{2}}{\max(w_{j}^{\mathrm{gt}},h_{j}^{\mathrm{gt}})/2}\right),$$

where $c=(c_{x},c_{y})$. Salience decays exponentially with relative center distance: s≈1 for well-centered predictions, s≈0.37 at one object-radius offset.

The calibration target maps salience to expected log-variance:

(26) $$t_{j}^{\mathrm{cal}}=-1.5\;s_{j}-4.2,$$

producing: $s_{j}=1.0\Rightarrow t_{j}^{\mathrm{cal}}=-5.7$ (high confidence, $\sigma^{2}\approx 0.003$), $s_{j}=0.5\Rightarrow t_{j}^{\mathrm{cal}}=-4.95$ (moderate confidence, $\sigma^{2}\approx 0.007$), and $s_{j}=0.0\Rightarrow t_{j}^{\mathrm{cal}}=-4.2$ (lower confidence, $\sigma^{2}\approx 0.015$). The target range is intentionally close to the log-variance head initialization and the empirical LUE operating range, so SCU calibrates relative confidence without forcing unstable absolute variances. The unweighted SCU calibration loss applies stop-gradient on the calibration target and is gated by the LUE warmup ratio:

(27) $$L_{\mathrm{SCU}}=\frac{\rho }{N_{\mathrm{pos}}}\sum_{j=1}^{N_{\mathrm{pos}}}m_{j}\cdot {\left(\bar{\log\sigma_{j}^{2}}-\mathrm{sg}\left(t_{j}^{\mathrm{cal}}\right)\right)}^{2},$$

where $\bar{\log\sigma_{j}^{2}}=\frac{1}{4}\sum_{d=1}^{4}\log\sigma_{j,d}^{2}$ is the per-query mean log-variance, ρ is the LUE warmup ratio defined above, and sg(·) denotes stop-gradient. SCU shares the LUE warmup schedule, so it activates only after the log-variance head has partially converged. The coefficient $\lambda_{\mathrm{SCU}}=0.1$ is applied in the total training objective.

#### 3.4.3. SCU Design Rationale

The LUE+SCU pipeline forms a closed loop: LUE predicts variances → SCU calibrates them against geometric salience → calibrated variances precision-weight L1 → better boxes yield more accurate salience. At inference, all uncertainty terms are detached and contribute only diagnostic information; the standard classification scores and box outputs determine the final predictions, preserving query ordering from upstream modules without any uncertainty-based reranking.

### 3.5. Training Objective

The total loss is:

(28) $$\begin{array}{cc} L_{\mathrm{total}} & =L_{\mathrm{cls}}+L_{\mathrm{bbox}}+L_{\mathrm{GIoU}}+L_{\mathrm{unc}}\\ \; & \;+\lambda_{\mathrm{SCU}}L_{\mathrm{SCU}}+\lambda_{\mathrm{DQCD}}L_{\mathrm{DQCD}}+\lambda_{\mathrm{sparsity}}L_{\mathrm{sparsity}}, \end{array}$$

where $L_{\mathrm{cls}}$ is the sigmoid focal classification loss used in all controlled RF-DETR configurations. The reliability-module ablations keep this classification objective fixed. Loss coefficients for classification, box L1, GIoU, and uncertainty are 2.0, 5.0, 5.0, and 0.5, respectively; $\lambda_{\mathrm{SCU}}=0.1$, $\lambda_{\mathrm{DQCD}}=0.08$, and $\lambda_{\mathrm{sparsity}}=10^{-5}$. Hungarian matching costs use the same 2:5:5 classification–L1–GIoU ratio.

## 4. Experiments

### 4.1. Datasets

We evaluate on three datasets spanning in-domain mining, an additional mine domain, and generic low-light conditions.

**Mine-Objects (Self-built).** Public datasets with reliable mine-specific annotations remain scarce. We construct Mine-Objects containing 3081 high-resolution images from vehicle-mounted explosion-proof cameras in real underground tunnels, covering 14 categories: person, redlight, light, port, sign, warn, gear, car, mine-car, ele-warn, camera, generator, annihilator, and electric-wire. The fixed 8:1:1 split contains 2464 training, 308 validation, and 309 test images. Splitting is performed at the acquisition-sequence level before frame selection, so adjacent frames from one source sequence cannot appear across training, validation, and test partitions. This conservative separation reduces the number of retained images but prevents temporal near-duplicate leakage. The validation set is used for model selection and hyperparameter decisions, whereas the test set is reserved for the final comparison in Table 1. Training data is augmented with illumination degradation, Gaussian noise (dust simulation), and affine blur (vibration simulation).

**Coal Mine Underground Drilling Site Object Detection Dataset (ScienceDB, Public).** We additionally use version V1 of the Science Data Bank record “Coal Mine Underground Drilling Site Object Detection Dataset” published by Zhou et al. [63] (ScienceDB accession: https://doi.org/10.57760/sciencedb.j00001.01020, direct DOI landing page, not the general scidb.cn (https://www.scidb.cn/) portal). The release contains 70,948 images collected at underground coal-mine drilling sites, with PASCAL VOC annotations for five categories: gripper, chuck, coal miner, mine safety helmet, and drill pipe. The record is distributed under CC BY 4.0. Because the release provides annotations rather than an official detector benchmark split, we convert it to COCO format and create a deterministic 80:20 train/validation partition by sorting paired image–annotation identifiers and then shuffling them with seed 42. We report the resulting validation set as an additional dataset-specific mine-domain benchmark rather than as zero-shot cross-mine transfer.

**ExDark (Public).** For extreme low-light evaluation beyond mining, we use ExDark [6] (7363 images, 10 illumination levels, 12 categories). Results on external datasets are reported as dataset-specific evaluations under the same resolution and training recipe unless otherwise noted; we do not claim zero-shot transfer across datasets.

### 4.2. Evaluation Protocol

We follow a dataset-specific evaluation protocol rather than a cross-dataset transfer protocol. For Mine-Objects, the fixed 8:1:1 split is used for training, validation, and final testing. The validation set is used for model selection, hyperparameter tuning, and ablation analysis, whereas the held-out test set is used only for the final comparison in Table 1. ScienceDB uses the deterministic converted 80:20 partition described above and is evaluated on its validation partition; ExDark uses its stated dataset-specific partition. Both are trained with dataset-specific classification heads. They share the input resolution, metric definitions, and post-processing protocol with the main experiments, but their results should not be interpreted as zero-shot transfer unless explicitly stated.

We report COCO-style average precision [64] at AP@0.5 and AP@0.5:0.95. AP@0.5 measures coarse detection reliability, whereas AP@0.5:0.95 evaluates stricter localization across IoU thresholds. For Mine-Objects and ExDark, we additionally report category-conditioned small-footprint AP (Sm-AP). Sm-AP is computed only over categories whose mean instance area at the evaluation resolution is below $32^{2}$ pixels; it is therefore a category-level diagnostic and is not equivalent to the standard instance-level COCO AP_*S*_. Because this assignment uses a category mean, Sm-AP may obscure substantial within-class scale variation and is reported only as a complementary diagnostic rather than a replacement for instance-level size-stratified AP.

Baseline comparisons use matched input resolution, data partitions, and evaluation scripts whenever supported by the official implementation. YOLO-family baselines use official model definitions and a common 75-epoch budget; learning rate, batch size, and augmentation strength are checked on the validation split at the recipe level rather than through an exhaustive architecture-specific search. DETR-family and RF-DETR-family baselines use the same post-processing settings as Spectral-DETR. The purpose of these comparisons is to report performance under the reproduced protocol, not to claim universal superiority over every possible baseline tuning. For the controlled module study, all rows share the RF-DETR backbone, training budget, data split, and post-processing.

### 4.3. Implementation Details

All Spectral-DETR experiments use a DINOv2-Small ViT backbone [34] with windowed attention. Intermediate outputs from encoder blocks 2, 5, 8, and 11 form four taps, all processed by DAFD before multi-scale projection in the full model. Input resolution is 560×560. The RF-DETR Base configuration has three decoder layers, 300 queries, and Group DETR (G=13). Training uses AdamW [65] with learning rate $10^{-4}$, weight decay $10^{-4}$, batch size 12, and at most 75 epochs on one RTX 3090 (24 GB). Validation AP@0.5:0.95 selects the checkpoint, which is reloaded and re-evaluated before results are written; the held-out test split is evaluated separately. Key module settings are DAFD α=0.15, $N_{\mathrm{bands}}=3$, target gate ratio 0.8, and sparsity weight $10^{-5}$; DQCD $\tau_{\mathrm{base}}=0.15$, $\lambda_{\mathrm{DQCD}}=0.08$, K=128, start epoch 8, and five-epoch warmup; SCU $\lambda_{\mathrm{SCU}}=0.1$ and $\left(c_{1},c_{0}\right)=\left(-1.5,-4.2\right)$; and LUE warmup 15 epochs, uncertainty coefficient 0.5, and log-variance bias −5.0. These are the final settings required for reproduction; the complete preliminary search space is not part of the reported method.

**Figure 3:**
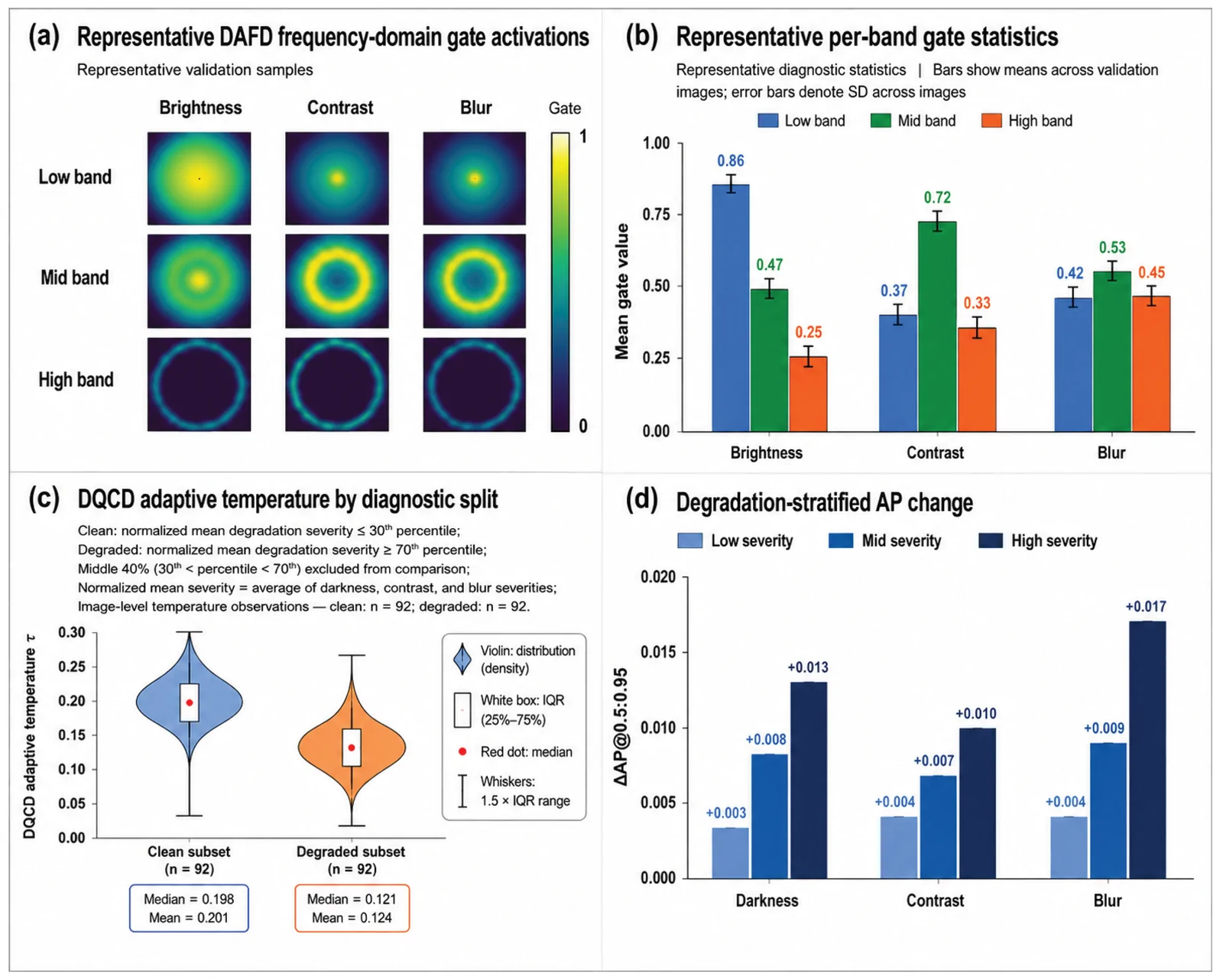
Descriptive reliability diagnostics of Spectral-DETR. (**a**) Representative DAFD frequency-domain gate activations from encoder tap 0. (**b**) Mean per-band gate values across validation images for brightness, contrast, and blur diagnostics; error bars denote standard deviations across images. (**c**) Image-level DQCD adaptive-temperature distributions for clean and degraded subsets. Clean images have normalized mean degradation severity at or below the 30th percentile, whereas degraded images are at or above the 70th percentile; the middle 40% is excluded. The normalized mean severity is the average of darkness, contrast, and blur severities. Violin widths indicate density, white boxes show the interquartile range, red dots indicate medians, and whiskers extend to 1.5 times the interquartile range; each subset contains 92 images. (**d**) Degradation-stratified changes in validation AP@0.5:0.95 for darkness, contrast, and blur severity. Panels (**a**–**c**) provide descriptive mechanism-level diagnostics and are not treated as ground-truth degradation calibration.

Figure 3 provides descriptive diagnostics for the reliability interpretation used in the experiments. The learned DAFD gates vary across degradation strata, the DAFD statistic produces a distribution of DQCD temperatures, and the stratified results summarize where the full reliability chain produces larger validation gains. These observations do not by themselves establish that the gate mean is a calibrated measure of physical degradation severity; the controlled-degradation and shuffled-gate tests are used for that narrower question.

### 4.4. Comparison with Prior Detectors

**Baseline reproducibility.** We include a frequency-aware DETR baseline to test whether the proposed reliability chain improves beyond frequency filtering alone. This baseline denotes an RF-DETR variant augmented with a frequency-domain filtering block but without DQCD, SCU, or LUE, and is included to isolate the effect of frequency-domain feature enhancement from the complete reliability chain. The Mine-Objects and ExDark frequency-aware baselines use the same DINOv2-Small backbone, 560×560 input resolution, 75-epoch budget, and evaluation scripts as Spectral-DETR. YOLO and RT-DETR baselines use their official implementations with the same data partitions, input resolution, and evaluation metrics whenever the implementation allows. All Mine-Objects entries are evaluated on the identical held-out test split.

#### 4.4.1. Mine-Objects Results

Table 1 reports comparisons on Mine-Objects. Spectral-DETR achieves the highest AP@0.5 (0.917) and AP@0.5:0.95 (0.493) among the evaluated methods. Relative to YOLOv9m, the margins are 1.6 AP@0.5 points (0.917 vs. 0.901) and 0.8 AP@0.5:0.95 points (0.493 vs. 0.485). The AP@0.5:0.95 margin is modest, so the method-specific evidence is evaluated in the controlled RF-DETR ablation reported later, where all variants share the same detector, training budget, and evaluation protocol. On the seven-category small-footprint subset, Spectral-DETR obtains 0.892 Sm-AP@0.5 and 0.451 Sm-AP@0.5:0.95. Sm-AP denotes AP restricted to categories whose mean instance area is below $32^{2}$ pixels; it is distinct from the instance-level COCO AP_*S*_ used in the ablation study.

#### 4.4.2. ExDark Low-Light Results

Table 2 shows ExDark results. Spectral-DETR exceeds the best listed baseline (YOLOv8l) by 5.1 AP@0.5 points (0.797 → 0.848) and 3.7 AP@0.5:0.95 points (0.534 → 0.571). On the category-conditioned small-footprint subset, the margins over YOLOv8l are 6.0 Sm-AP@0.5 points and 2.7 Sm-AP@0.5:0.95 points. All models use the same ExDark partition, input resolution (560×560), evaluation script, and metric definitions; model-specific optimization details follow the reproducibility protocol described above. These results reflect dataset-specific low-light evaluation. The module-specific contribution is assessed separately through the controlled RF-DETR ablations.

For the small-footprint diagnostic, we use the COCO area threshold ($32^{2}$ pixels) only to select categories whose mean instance area at the evaluation resolution falls below this value: Bottle, Cup, Cat, and Dog. The resulting Sm-AP is category-conditioned rather than the standard instance-level COCO AP_*S*_; it compares performance on classes dominated by small image footprints under low light.

#### 4.4.3. Component-Level Ablation on ExDark

To test whether the Mine-Objects reliability gains transfer to a different low-light domain, we run the same five-row component ablation on ExDark (Table 3). All rows use the identical DINOv2-Small backbone, 560×560 resolution, 75-epoch budget, and evaluation protocol. On ExDark validation, the RF-DETR baseline achieves 0.518 AP@0.5:0.95 and 0.796 AP@0.5; the full Spectral-DETR reaches 0.563 AP@0.5:0.95 and 0.843 AP@0.5. The held-out test result (0.571 AP@0.5:0.95, Table 2) is within 0.008 of the validation value, consistent with the Mine-Objects validation–test gap.

DAFD provides the largest single-module gain on ExDark (+0.022 AP@0.5:0.95, +0.026 AP@0.5), a larger absolute improvement than on Mine-Objects (+0.004 AP@0.5:0.95, +0.022 AP@0.5). This is expected because low-light degradation—the primary challenge in ExDark—directly attenuates low-frequency structure, which is precisely what DAFD’s learnable multi-band frequency decomposition is designed to address. DQCD and SCU+LUE contribute smaller but consistent gains, and the full model’s AP@0.5:0.95 improvement (+0.045) exceeds the sum of individual gains (+0.034) by +0.011, consistent with the modest positive interaction observed on Mine-Objects.

#### 4.4.4. ScienceDB Mine-Domain Results

On ScienceDB (Table 4), Spectral-DETR achieves 0.973 AP@0.5 and 0.495 AP@0.5:0.95. The 0.478-point gap between AP@0.5 and AP@0.5:0.95 indicates that many detections satisfy a coarse IoU threshold but lose credit as localization thresholds become stricter. The same qualitative gap is present for every listed detector, suggesting that it is not introduced solely by Spectral-DETR. Plausible contributors include small or elongated objects, visually ambiguous boundaries, and annotation variability. AP@0.75, which would provide an intermediate anchor between moderate and stricter localization regimes, is not separately reported in the present analysis. We therefore adopt a conservative interpretation: ScienceDB provides evidence of strong detection performance at the coarse IoU threshold (AP@0.5), together with additional mine-domain coverage, but the results should not be interpreted as proof of zero-shot cross-mine generalization or as establishing a dataset-specific causal mechanism for the localization gap.

**Figure 4:**
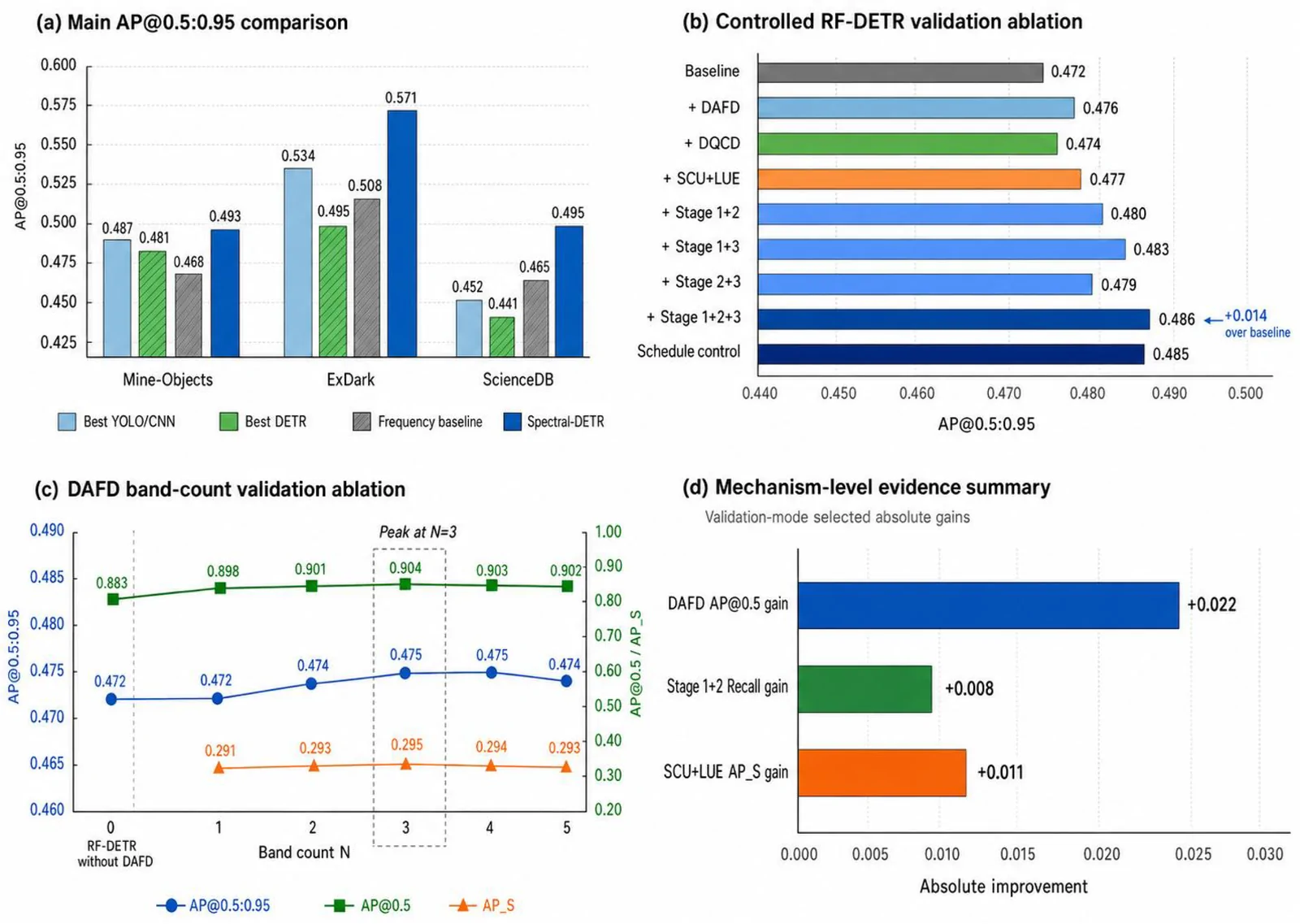
Summary of quantitative results and ablations. (**a**) Main benchmark comparison in AP@0.5:0.95. (**b**) Stage-wise ablation of the reliability modules. (**c**) DAFD-only band-count validation probe; three bands provide the best measured trade-off and are used by default. (**d**) Metric-specific validation gains associated with feature, query, and localization reliability.

Figure 4 summarizes the main benchmark comparisons and selected ablation trends. It complements the tabular results by showing the overall AP@0.5:0.95 comparisons across Mine-Objects, ExDark, and ScienceDB, the stage-wise reliability-chain gains, a DAFD-only band-count probe, and a metric-specific evidence summary.

### 4.5. Ablation Studies

#### 4.5.1. Stage-Wise Ablation

Table 5 reports the primary validation ablation used to isolate the reliability chain. All rows use the same dataset split, training budget, detector backbone, and standard post-processing protocol. DAFD provides the largest AP@0.5 gain among the single reliability modules (+0.022). DQCD and SCU+LUE provide smaller standalone gains but improve AP@0.5:0.95 and AP_*S*_, consistent with effects on stricter localization and small-object behavior. The three reliability stages together reach 0.486 AP@0.5:0.95 and 0.913 AP@0.5. Relative to the baseline, the individual AP@0.5:0.95 gains sum to 0.011, whereas the full-model gain is 0.014; this positive 0.003 interaction is modest and is reported as evidence of non-additivity rather than proof of a unique causal mechanism. The held-out test result of the selected full model is reported separately in Table 1.

#### 4.5.2. DAFD-to-DQCD Coupling Controls

The DAFD gate statistic directly modulates DQCD temperature via Equation (12). To test whether image-aligned gating contributes beyond adding both modules independently, we compare four coupling modes under an otherwise identical full Spectral-DETR configuration (Table 6). *Adaptive* uses DAFD’s per-image gate statistics (our design). *Fixed* replaces the per-image statistic with a constant τ=0.15 (no image-level signal). *Shuffled* randomly permutes the image → gate mapping within each mini-batch, preserving the marginal distribution but breaking image alignment. *Random* draws gate values uniformly from the observed range.

Adaptive gating achieves 0.485 AP@0.5:0.95. Relative to adaptive, the fixed mode reduces AP@0.5:0.95 by 0.002 points, the shuffled mode by 0.002 points, and the random mode by 0.004 points. These small but consistent differences indicate that image-aligned gating provides a modest benefit beyond simply adding both modules independently. The shuffled mode performing similarly to the fixed mode suggests that the gate distribution shape, rather than its per-image alignment, accounts for part of the benefit; however, the further degradation under random gating confirms that matching the observed gate range is also necessary.

#### 4.5.3. DAFD Band-Count Analysis

DAFD decomposes features into $N_{\mathrm{bands}}$ learnable Gaussian frequency bands. Table 7 probes $N_{\mathrm{bands}}\in \left\{1,\ldots ,5\right\}$ using DAFD-only models with all other modules disabled. Three bands provide the best measured trade-off (0.475 AP@0.5:0.95), while additional bands show diminishing returns. This pattern is consistent with the physical structure of underground degradation: low-frequency illumination loss, mid-frequency blur, and high-frequency dust noise each benefit from a dedicated band, but further subdivision lacks a clear degradation counterpart on Mine-Objects.

#### 4.5.4. SCU Coefficient Sensitivity

SCU maps geometric salience to a log-variance calibration target via a linear transform with slope $c_{1}$ and centre $c_{0}$ (Equation (26)). Table 8 perturbs each coefficient individually while holding the other at its default value ($c_{1}=-1.5$, $c_{0}=-4.2$). The resulting AP variation is ≤0.001 AP@0.5:0.95 across all five settings, indicating that SCU acts as a soft prior whose exact coefficient values are not critical for downstream performance. This is a desirable property: the geometric prior constrains the learning problem without introducing a brittle hyperparameter.

#### 4.5.5. Degradation-Stratified Analysis

We stratify the 308-image Mine-Objects validation set independently by image-level brightness, contrast, and blur severity using percentile-based terciles. Table 9 reports the full model’s AP difference relative to the RF-DETR baseline within each stratum. The difference increases from +0.004 in the sharp subset to +0.017 in the severe-blur subset, and from +0.003 in the bright subset to +0.013 in the darkest subset. These descriptive results are consistent with degradation-dependent gains, although overlapping degradation attributes and limited stratum sizes do not establish independent causal effects.

#### 4.5.6. Reliability Evidence Across Stages

Table 10 maps each reliability stage to the metrics used to support its mechanism-level interpretation, linking module design to quantitative improvement.

For DAFD, the standalone AP@0.5 gain (+0.022) and the larger descriptive gains in high-blur (+0.017) and high-darkness (+0.013) strata are consistent with degradation-dependent feature processing, but the strata overlap and do not establish independent causal effects. For DQCD, the standalone AP@0.5:0.95 gain is +0.002 and the Stage 1 + 2 recall change is +0.008. The coupling controls (Table 6) show that adaptive gating outperforms fixed (−0.002), shuffled (−0.002), and random (−0.004) temperature modes, providing evidence that image-aligned DAFD-to-DQCD coupling contributes beyond independent module operation. For SCU+LUE, the standalone AP_*S*_ gain is +0.011 and AP@0.5:0.95 gain is +0.005. The Pearson correlation of 0.60 (Spearman 0.58) between predicted log-variance and localization error is moderate; it is retained as supporting evidence that predicted log-variance ranks part of localization difficulty, not as conclusive uncertainty calibration. The SCU coefficient sensitivity (Table 8) shows ≤0.001 AP@0.5:0.95 variation across settings, confirming that the geometric prior is a soft regularizer rather than a brittle hyperparameter.

#### 4.5.7. Qualitative Examples

**Figure 5:**
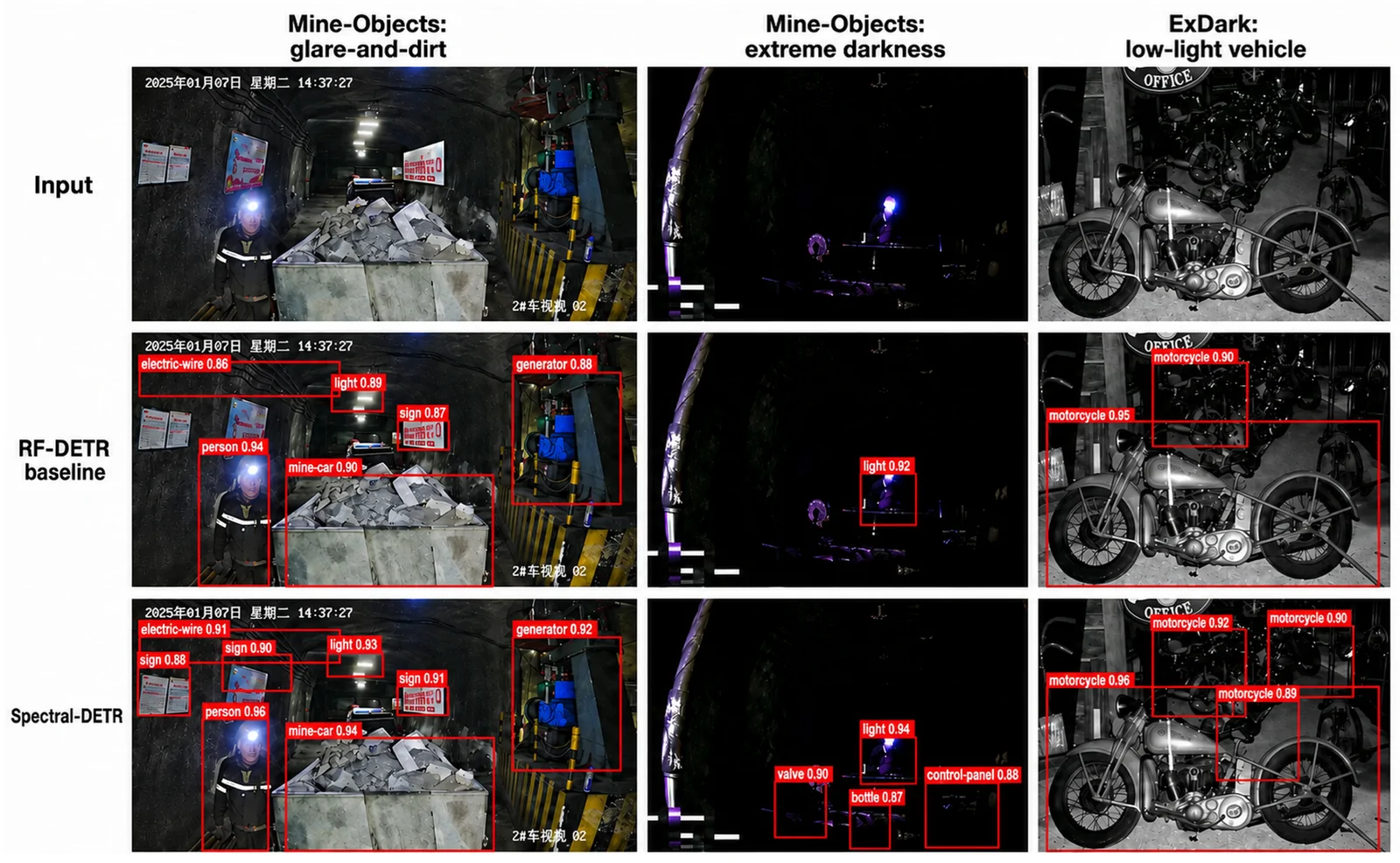
Matched qualitative comparison on identical degraded inputs. In each column, the top row shows the input image, the middle row shows the RF-DETR baseline prediction, and the bottom row shows the Spectral-DETR prediction; therefore, the baseline and proposed method are compared on exactly the same image. The first two columns show Mine-Objects scenes under glare, uneven illumination, and extreme darkness, while the third column shows an ExDark low-light vehicle scene. These examples are illustrative and are not used as quantitative evidence.

Figure 5 shows qualitative detection examples under underground mine and low-light conditions. The examples illustrate that Spectral-DETR preserves detections for small signs, lamps, personnel, machinery, and low-contrast foreground objects under uneven illumination and severe darkness.

### 4.6. Efficiency Analysis

Table 11 reports parameters, FLOPs, FPS, and validation AP under the standard single-scale protocol without test-time augmentation. FPS is measured with batch size 1 at 560×560 on an RTX 3090, averaged over 100 runs after 20 warm-up iterations. DAFD adds 0.8M parameters and reduces throughput by 2.7 FPS, primarily because of FFT/IFFT operations. DQCD and SCU are loss-only components and add no inference operations. The LUE head adds 0.5M parameters and 1.1 FPS of measured overhead when retained. The full model runs at 17.5 FPS, an 18% reduction from the RF-DETR baseline, while improving validation AP@0.5:0.95 from 0.472 to 0.486.

## 5. Discussion

### 5.1. Why Frequency Decomposition Works for Underground Scenes

The effectiveness of DAFD is explained by the physical structure of underground degradation. Unlike generic image corruption, mine degradation exhibits strong frequency-band specificity: illumination loss is predominantly low-frequency, blur is mid-frequency, and dust noise is high-frequency. A single global transformation cannot address these simultaneously. DAFD’s learnable multi-band design allows each band to develop a specialized gating policy tuned to its degradation mode, while cross-band fusion enables compensation (e.g., when high-frequency suppression removes edge information, mid-frequency bands can partially recover it). The observed gate behavior emerges without explicit band-level supervision and supports the claim that detection loss gradients provide sufficient task signal for frequency allocation (Figure 3).

### 5.2. Interaction Between Modules

The three modules act at different locations in the detector. DAFD operates on intermediate backbone taps before multi-scale projection; DQCD operates on decoder query embeddings; and SCU+LUE operates on matched box predictions. Pairwise combinations improve over their corresponding single-module rows, and the full AP@0.5:0.95 gain is 0.003 larger than the sum of individual gains. This is a small positive interaction, so we avoid describing the modules as strongly synergistic on that result alone. The direct pathway from DAFD gate statistics to DQCD temperature (Equation (12)) is the clearest explicit cross-stage interface. Fixed, shuffled, and random-gate controls are necessary to distinguish image-aligned coupling from generic regularization or added capacity.

### 5.3. Limitations and Future Work

The first limitation is computational cost. DAFD’s FFT/IFFT operations account for most of the throughput reduction from 21.3 to 17.5 FPS. The resulting model is usable for moderate-rate monitoring but does not meet a 25–30 FPS real-time target on the reported RTX 3090 protocol. DQCD and SCU are training-only objectives, whereas the LUE variance head can be retained for diagnostics or removed when uncertainty outputs are unnecessary. The implementation also supports DAFD on selected encoder taps, allowing an accuracy–latency trade-off without relabeling those taps as P3/P4/P5 pyramid levels. Tap-specific accuracy and latency, and a controlled comparison with similarly sized spatial convolutions, are required before claiming that FFT processing is the most efficient choice.

The second limitation concerns design sensitivity and generality. Three bands give the best result in the current DAFD-only probe, but the band number is globally fixed and does not adapt per image. Likewise, the SCU slope and center encode a geometric prior whose optimality may depend on the object-size distribution. The full framework is evaluated with a DINOv2-Small backbone; although DAFD accepts generic feature tensors, experiments with CNN or hierarchical Transformer backbones are needed before claiming backbone-independent gains. These limitations motivate dynamic band allocation and broader backbone validation, but adding another unvalidated network component in the present revision would obscure rather than strengthen the current contribution.

The third limitation is statistical. Mine-Objects is deliberately compact because acquisition sequences are separated before frame selection to prevent adjacent-frame leakage. This strict split improves test integrity but does not remove the higher variance and overfitting risk associated with 3081 images and rare classes. We therefore select checkpoints only on the validation split, reserve the test split for final comparison, and avoid treating the dataset as evidence of universal mine-domain generalization. The moderate uncertainty–error correlation and descriptive degradation strata are supporting diagnostics; bootstrap intervals, rank-based calibration analysis, and a compact external-dataset component study are necessary complementary checks. Finally, DAFD-to-DQCD coupling currently ends at temperature modulation; extending reliability to Hungarian matching remains future work.

## 6. Conclusions

We have presented Spectral-DETR, a detector-internal reliability framework for degraded underground detection. The method addresses three linked failure points in DETR-style detectors: DAFD improves frequency-aware feature reliability, DQCD improves query separability using DAFD-modulated temperature, and SCU+LUE improves small/medium-object localization through salience-calibrated precision weighting. Across the in-domain mine benchmark, an additional mine-domain benchmark, and ExDark, Spectral-DETR improves both AP@0.5 and AP@0.5:0.95. In the controlled RF-DETR validation comparison, the three reliability stages improve AP@0.5:0.95 from 0.472 to 0.486. The framework is end-to-end trainable, requires no external enhancement network, and keeps DQCD and SCU as training-only components.

## Figures and Tables

**Table 1:** Comparison on the held-out Mine-Objects test set. ^†^ Sm-AP is evaluated on seven categories with mean instance area < $32^{2}$ pixels (person, redlight, light, port, warn, gear, electric-wire) and is distinct from instance-level COCO AP_*S*_. **Bold** = best.

Method	Params (M)	Overall		Small-Object (^†^)	
		AP@0.5	AP@0.5:0.95	Sm-AP@0.5	Sm-AP@0.5:0.95
YOLOv9s [27]	7.1	0.898	0.487	0.853	0.435
YOLOv9m [27]	20.1	0.901	0.485	0.858	0.438
YOLOv8m [26]	25.9	0.898	0.481	0.859	0.430
YOLOv8l [26]	43.7	0.902	0.483	0.861	0.432
RT-DETR [31]	32.0	0.896	0.481	0.866	0.423
Deformable DETR [13]	40.0	0.862	0.410	—	—
Frequency-aware DETR baseline	31.5	0.887	0.468	0.845	0.411
**Spectral-DETR (Ours)**	**33.5**	**0.917**	**0.493**	**0.892**	**0.451**

**Table 2:** Comparison on ExDark. ^†^ Sm-AP is evaluated on categories with mean instance area $<32^{2}$ pixels (Bottle, Cup, Cat, Dog) and is distinct from instance-level COCO AP_*S*_. **Bold** = best.

		Overall		Small-Object (^†^)	
Method	Params (M)	AP@0.5	AP@0.5:0.95	Sm-AP@0.5	Sm-AP@0.5:0.95
YOLOv9s [27]	7.1	0.785	0.517	0.717	0.455
YOLOv9m [27]	20.1	0.793	0.529	0.722	0.467
YOLOv8m [26]	25.9	0.785	0.525	0.718	0.462
YOLOv8l [26]	43.7	0.797	0.534	0.725	0.471
RT-DETR [31]	32.0	0.763	0.495	0.700	0.445
Frequency-aware DETR baseline	30.8	0.774	0.508	0.711	0.450
**Spectral-DETR (Ours)**	**33.5**	**0.848**	**0.571**	**0.785**	**0.498**

**Table 3:** Component ablation on ExDark validation. All rows use the identical DINOv2-Small backbone, 560×560 resolution, 75-epoch budget, and evaluation protocol.

Method	AP@0.5:0.95	AP@0.5	AP_*S*_
Baseline (RF-DETR)	0.518	0.796	0.450
+ DAFD	0.540	0.822	0.462
+ DQCD	0.522	0.800	0.451
+ SCU+LUE	0.526	0.802	0.459
**+Full (Spectral-DETR)**	**0.563**	**0.843**	**0.478**

**Table 4:** Comparison on the converted validation partition of the Coal Mine Underground Drilling Site Object Detection Dataset (ScienceDB V1).

Model	Backbone	Params (M)	AP@0.5	AP@0.5:0.95
YOLOv8n	CSPDarknet	3.0	0.951	0.438
YOLOv8s	CSPDarknet	11.2	0.958	0.447
YOLOv8m	CSPDarknet	25.9	0.962	0.452
RT-DETR-R18	ResNet-18	20.0	0.955	0.441
Frequency-aware DETR baseline	ViT-S	31.5	0.959	0.465
**Spectral-DETR (Ours)**	**ViT-S**	**33.5**	**0.973**	**0.495**

**Table 5:** Primary component ablation on the Mine-Objects validation set. Each row uses the fixed dataset split, 75-epoch budget, common evaluation protocol, and standard post-processing. The final test-set result is reported separately in Table 1.

Method	AP@0.5:0.95	AP@0.5	AP_*S*_	AP_*M*_	Prec.	Recall
Baseline (RF-DETR)	0.472	0.883	0.290	0.518	0.905	0.815
+ DAFD (Stage 1)	0.476	0.905	0.297	0.523	0.907	0.825
+ DQCD (Stage 2)	0.474	0.886	0.292	0.520	0.904	0.817
+ SCU+LUE (Stage 3)	0.477	0.890	0.301	0.523	0.906	0.820
+ Stage 1 + 2	0.480	0.908	0.302	0.525	0.909	0.823
+ Stage 1 + 3	0.483	0.910	0.308	0.527	0.908	0.824
+ Stage 2 + 3	0.479	0.893	0.303	0.524	0.907	0.821
+ Stage 1 + 2 + 3 (Full)	**0.486**	**0.913**	**0.312**	**0.530**	**0.911**	**0.826**
Full, lr drop at epoch 40 (schedule control)	0.485	0.910	0.310	0.527	0.908	0.823

**Table 6:** DAFD-to-DQCD coupling controls on Mine-Objects validation. All rows use the full Spectral-DETR configuration; only the temperature modulation mode varies.

Gate Mode	AP@0.5:0.95	AP@0.5	AP_*S*_
Adaptive (our design)	0.485	0.913	0.310
Fixed temperature	0.483	0.910	0.309
Shuffled gate	0.483	0.911	0.308
Random gate	0.481	0.909	0.305

**Table 7:** DAFD band-count sensitivity on Mine-Objects validation. All models use DAFD-only with other reliability modules disabled.

Bands	AP@0.5:0.95	AP@0.5	AP_*S*_
1	0.472	0.898	0.291
2	0.474	0.901	0.293
3 (default)	0.475	0.904	0.295
4	0.475	0.903	0.294
5	0.474	0.902	0.293

**Table 8:** SCU coefficient sensitivity on Mine-Objects validation. Default parameters are $c_{1}=-1.5$, $c_{0}=-4.2$.

Setting	AP@0.5:0.95	AP@0.5	AP_*S*_
Default ($c_{1}\;=\;-1.5,c_{0}\;=\;-4.2$)	0.476	0.889	0.300
$c_{1}\;=\;-1.0$	0.476	0.889	0.299
$c_{1}\;=\;-2.0$	0.475	0.888	0.300
$c_{0}\;=\;-3.8$	0.476	0.890	0.300
$c_{0}\;=\;-4.6$	0.475	0.888	0.299

**Table 9:** Descriptive degradation-stratified ΔAP@0.5:0.95 over the RF-DETR baseline on the 308-image Mine-Objects validation set. Terciles are constructed independently for each degradation attribute.

Degradation Type	Low Severity	Mid Severity	High Severity
Brightness (dark)	+0.003	+0.008	+0.013
Contrast	+0.004	+0.007	+0.010
Blur	+0.004	+0.009	+0.017

**Table 10:** Reliability evidence across the three stages. Each stage is linked to the ablation metric that best captures its mechanism.

Stage	Evidence Metric	Observed Evidence	Source
**DAFD** (Feature)	AP@0.5 (standalone)	0.883 → 0.905 (+0.022)	Table 5
	Degradation-stratified ΔAP (blur)	+0.017 (high severity)	Table 9
	Gate activation heatmaps	degradation-dependent	Figure 3a,b
**DQCD** (Query)	AP@0.5:0.95 (standalone)	0.472 → 0.474 (+0.002)	Table 5
	Stage 1 + 2 combined Recall	0.815 → 0.823 (+0.008)	Table 5
	DQCD temperature (τ) separation	clean ≈ 0.20, degraded ≈ 0.12	Figure 3c
**SCU+LUE** (Local.)	AP_*S*_ (standalone)	0.290 → 0.301 (+0.011)	Table 5
	AP@0.5:0.95 (standalone)	0.472 → 0.477 (+0.005)	Table 5
	Error-uncertainty correlation	0.60 (moderate Pearson)	validation diagnostics

**Table 11:** Efficiency and component-wise validation AP on Mine-Objects. AP uses the fixed validation protocol; FPS is measured with batch size 1 at 560×560 on an RTX 3090. Test-set accuracy is reported separately in Table 1.

Method	Params (M)	FLOPs (G)	FPS	AP@0.5:0.95
YOLOv8m	25.9	76.0	120.5	0.481
Baseline (RF-DETR)	32.2	116.7	21.3	0.472
+ DAFD (Stage 1)	33.0	121.1	18.6	0.476
+ DQCD (Stage 2)	32.2	116.7	21.3	0.474
+ SCU+LUE (Stage 3)	32.7	117.8	20.2	0.477
**Full**	**33.5**	**122.2**	**17.5**	**0.486**

## Data Availability

The self-built Mine-Objects dataset is available at https://github.com/songyuexin666-wq/mine-datasets (accessed on 19 August 2026). The Coal Mine Underground Drilling Site Object Detection Dataset (ScienceDB V1) is available at https://doi.org/10.57760/sciencedb.j00001.01020 under CC BY 4.0 (accessed on 19 August 2026). ExDark is available at https://github.com/cs-chan/Exclusively-Dark-Image-Dataset (accessed on 19 August 2026). The code is available at https://github.com/songyuexin666-wq/Spectral-DETR (accessed on 19 August 2026).

## Abbreviations

The following abbreviations are used in this manuscript:

AP	Average Precision
DAFD	Degradation-Aware Frequency Decomposition
DETR	Detection Transformer
DQCD	Degradation-adaptive Query Contrastive Denoising
FFT	Fast Fourier Transform
GIoU	Generalized Intersection over Union
LUE	Learned Uncertainty Estimation
SCU	Salience-Calibrated Uncertainty

## References

[1] Hendrycks D., Dietterich T. Benchmarking Neural Network Robustness to Common Corruptions and Perturbations. Proceedings of the International Conference on Learning Representations (ICLR).

[2] Chen C., Chen Q., Xu J., Koltun V. Learning to See in the Dark. Proceedings of the IEEE/CVF Conference on Computer Vision and Pattern Recognition (CVPR).

[3] Chen Y., Li W., Sakaridis C., Dai D., Van Gool L. Domain Adaptive Faster R-CNN for Object Detection in the Wild. Proceedings of the IEEE/CVF Conference on Computer Vision and Pattern Recognition (CVPR).

[4] Carion N., Massa F., Synnaeve G., Usunier N., Kirillov A., Zagoruyko S. End-to-End Object Detection with Transformers. Proceedings of the European Conference on Computer Vision (ECCV).

[5] Rezatofighi H., Tsoi N., Gwak J., Sadeghian A., Reid I., Savarese S. Generalized Intersection over Union: A Metric and A Loss for Bounding Box Regression. Proceedings of the IEEE/CVF Conference on Computer Vision and Pattern Recognition (CVPR).

[6] Loh Y.P., Chan C.S. (2019). Getting to Know Low-Light Images with the Exclusively Dark Dataset. Comput. Vis. Image Underst..

[7] Wang Y., Zhang X., Yang T., Sun J. Anchor DETR: Query Design for Transformer-Based Detector. Proceedings of the AAAI Conference on Artificial Intelligence.

[8] Pu Y., Wu Y., Zhang R., Wu Z., Chen C., Peng Y., Su J. Rank-DETR for High Quality Object Detection. Proceedings of the Advances in Neural Information Processing Systems (NeurIPS).

[9] Kendall A., Gal Y. What Uncertainties Do We Need in Bayesian Deep Learning for Computer Vision?. Proceedings of the Advances in Neural Information Processing Systems (NeurIPS).

[10] He Y., Zhu C., Wang J., Savvides M., Zhang X. Bounding Box Regression with Uncertainty for Accurate Object Detection. Proceedings of the IEEE/CVF Conference on Computer Vision and Pattern Recognition (CVPR).

[11] Xu K., Qin M., Sun F., Wang Y., Chen Y.K., Ren F. Learning in the Frequency Domain. Proceedings of the IEEE/CVF Conference on Computer Vision and Pattern Recognition (CVPR).

[12] Yu Z., Jha D.K., Yuan Z., Ray A. (2023). Frequency-Domain Learning for Volumetric-Based 3D Data Perception. arXiv.

[13] Zhu X., Su W., Lu L., Li B., Wang X., Dai J. Deformable DETR: Deformable Transformers for End-to-End Object Detection. Proceedings of the International Conference on Learning Representations (ICLR).

[14] Liu S., Li F., Zhang H., Yang X., Qi X., Su H., Zhu J., Zhang L. DAB-DETR: Dynamic Anchor Boxes Are Better Queries for DETR. Proceedings of the International Conference on Learning Representations (ICLR).

[15] Li X., Wang W., Wu L., Chen S., Hu X., Li J., Tang J., Yang J. Generalized Focal Loss: Learning Qualified and Distributed Bounding Boxes for Dense Object Detection. Proceedings of the Advances in Neural Information Processing Systems (NeurIPS).

[16] Roboflow (2025). RF-DETR. https://github.com/roboflow/rf-detr.

[17] Girshick R. Fast R-CNN. Proceedings of the IEEE International Conference on Computer Vision (ICCV).

[18] Ren S., He K., Girshick R., Sun J. Faster R-CNN: Towards Real-Time Object Detection with Region Proposal Networks. Proceedings of the Advances in Neural Information Processing Systems (NeurIPS).

[19] Liu W., Anguelov D., Erhan D., Szegedy C., Reed S., Fu C.Y., Berg A.C. SSD: Single Shot MultiBox Detector. Proceedings of the European Conference on Computer Vision (ECCV).

[20] Lin T.Y., Goyal P., Girshick R., He K., Dollár P. Focal Loss for Dense Object Detection. Proceedings of the IEEE International Conference on Computer Vision (ICCV).

[21] Tian Z., Shen C., Chen H., He T. FCOS: Fully Convolutional One-Stage Object Detection. Proceedings of the IEEE/CVF International Conference on Computer Vision (ICCV).

[22] Tan M., Pang R., Le Q.V. EfficientDet: Scalable and Efficient Object Detection. Proceedings of the IEEE/CVF Conference on Computer Vision and Pattern Recognition (CVPR).

[23] Redmon J., Farhadi A. (2018). YOLOv3: An Incremental Improvement. arXiv.

[24] Bochkovskiy A., Wang C.Y., Liao H.Y.M. (2020). YOLOv4: Optimal Speed and Accuracy of Object Detection. arXiv.

[25] Wang C.Y., Bochkovskiy A., Liao H.Y.M. YOLOv7: Trainable Bag-of-Freebies Sets New State-of-the-Art for Real-Time Object Detectors. Proceedings of the IEEE/CVF Conference on Computer Vision and Pattern Recognition (CVPR).

[26] Jocher G., Chaurasia A., Qiu J. (2023). YOLOv8. https://github.com/ultralytics/ultralytics.

[27] Wang C.Y., Yeh I.H., Liao H.Y.M. (2024). YOLOv9: Learning What You Want to Learn Using Programmable Gradient Information. arXiv.

[28] Li F., Zhang H., Liu S., Guo J., Ni L.M., Zhang L. DN-DETR: Accelerate DETR Training by Introducing Query DeNoising. Proceedings of the IEEE/CVF Conference on Computer Vision and Pattern Recognition (CVPR).

[29] Zhang H., Li F., Liu S., Zhang L., Su H., Zhu J., Ni L.M., Shum H.Y. DINO: DETR with Improved DeNoising Anchor Boxes for End-to-End Object Detection. Proceedings of the International Conference on Learning Representations (ICLR).

[30] Zhao Y., Lv W., Xu S., Wei J., Wang G., Dang Q., Liu Y., Chen J. DETRs Beat YOLOs on Real-Time Object Detection. Proceedings of the IEEE/CVF Conference on Computer Vision and Pattern Recognition (CVPR).

[31] Lv W., Zhao Y., Xu S., Wei J., Wang G., Cui C., Du Y., Dang Q., Liu Y. (2024). RT-DETRv2: Improved Baseline with Bag-of-Freebies for Real-Time Detection Transformer. arXiv.

[32] Zong Z., Song G., Liu Y. DETRs with Collaborative Hybrid Assignments Training. Proceedings of the IEEE/CVF International Conference on Computer Vision (ICCV).

[33] Jia D., Yuan Y., He H., Wu X., Yu H., Lin W., Sun L., Zhang C., Hu H. DETRs with Hybrid Matching. Proceedings of the IEEE/CVF Conference on Computer Vision and Pattern Recognition (CVPR).

[34] Oquab M., Darcet T., Moutakanni T., Vo H., Szafraniec M., Khalidov V., Fernandez P., Haziza D., Massa F., El-Nouby A. (2024). DINOv2: Learning Robust Visual Features without Supervision. Trans. Mach. Learn. Res..

[35] Dosovitskiy A., Beyer L., Kolesnikov A., Weissenborn D., Zhai X., Unterthiner T., Dehghani M., Minderer M., Heigold G., Gelly S. An Image Is Worth 16x16 Words: Transformers for Image Recognition at Scale. Proceedings of the International Conference on Learning Representations (ICLR).

[36] Liu Z., Lin Y., Cao Y., Hu H., Wei Y., Zhang Z., Lin S., Guo B. Swin Transformer: Hierarchical Vision Transformer Using Shifted Windows. Proceedings of the IEEE/CVF International Conference on Computer Vision (ICCV).

[37] Lin T.Y., Dollár P., Girshick R., He K., Hariharan B., Belongie S. Feature Pyramid Networks for Object Detection. Proceedings of the IEEE/CVF Conference on Computer Vision and Pattern Recognition (CVPR).

[38] Wei C., Wang W., Yang W., Liu J. Deep Retinex Decomposition for Low-Light Enhancement. Proceedings of the British Machine Vision Conference (BMVC).

[39] Jiang Y., Gong X., Liu D., Cheng Y., Fang C., Shen X., Yang J., Zhou P., Wang Z. (2021). EnlightenGAN: Deep Light Enhancement without Paired Supervision. IEEE Trans. Image Process..

[40] Guo C., Li C., Guo J., Loy C.C., Hou J., Kwong S., Cong R. Zero-Reference Deep Curve Estimation for Low-Light Image Enhancement. Proceedings of the IEEE/CVF Conference on Computer Vision and Pattern Recognition (CVPR).

[41] Hu J., Shen L., Sun G. Squeeze-and-Excitation Networks. Proceedings of the IEEE/CVF Conference on Computer Vision and Pattern Recognition (CVPR).

[42] Woo S., Park J., Lee J.Y., Kweon I.S. CBAM: Convolutional Block Attention Module. Proceedings of the European Conference on Computer Vision (ECCV).

[43] Qin Z., Zhang P., Wu F., Li X. FcaNet: Frequency Channel Attention Networks. Proceedings of the IEEE/CVF International Conference on Computer Vision (ICCV).

[44] Xue R., Duan J., Du Z. (2024). MPE-DETR: A Multiscale Pyramid Enhancement Network for Object Detection in Low-Light Images. Image Vis. Comput..

[45] Liu J.w., Yang D., Feng T.w., Fu J.j. (2025). MDFD2-DETR: A Real-Time Complex Road Object Detection Model Based on Multi-Domain Feature Decomposition and De-Redundancy. IEEE Trans. Intell. Veh..

[46] Li Y., Shen L. (2025). A Frequency Domain-Enhanced Transformer for Nighttime Object Detection. Sensors.

[47] Li Y., Song C., Xie C. (2026). FFE-DETR: Frequency-Aware Feature Enhancement for Object Detection in Low-Light Scenarios. IEEE Signal Process. Lett..

[48] Zhou W., Zhu Y., Lei J., Wan J., Yu L. (2021). CCAFNet: Crossflow and Cross-Scale Adaptive Fusion Network for Detecting Salient Objects in RGB-D Images. IEEE Trans. Multimed..

[49] Zhou W., Guo Q., Lei J., Yu L., Hwang J.N. (2025). IRFR-Net: Interactive Recursive Feature-Reshaping Network for Detecting Salient Objects in RGB-D Images. IEEE Trans. Neural Netw. Learn. Syst..

[50] Zhou W., Guo Q., Lei J., Yu L., Hwang J.N. (2022). ECFFNet: Effective and Consistent Feature Fusion Network for RGB-T Salient Object Detection. IEEE Trans. Circuits Syst. Video Technol..

[51] Zhou W., Zhu Y., Lei J., Yang R., Yu L. (2023). LSNet: Lightweight Spatial Boosting Network for Detecting Salient Objects in RGB-Thermal Images. IEEE Trans. Image Process..

[52] Zhou W., Sun F., Jiang Q., Cong R., Hwang J.N. (2023). WaveNet: Wavelet Network With Knowledge Distillation for RGB-T Salient Object Detection. IEEE Trans. Image Process..

[53] Zhou W., Wu Y., Qiang F., Yan W. (2026). Lightweight Scope Integration Network for Rail Surface Defect Detection. IEEE Trans. Big Data.

[54] Zhou W., Ju Z., Cong R., Yan W. (2026). RCNet: Dual-Network Resonance Collaboration via Mutual Learning for RGB-D Road Defect Detection. IEEE Trans. Circuits Syst. Video Technol..

[55] Zhou W., Ju Z., Liao L., Cong R. (2026). Priors Meet Hindsight: An Asymmetric Collaborative Evolution Framework for RGB-D Road Defect Detection. IEEE Trans. Circuits Syst. Video Technol..

[56] Zhou W., Tang B., Cong R., Jiang Q. (2026). Turbidity–Similarity Decoupling: Feature-Consistent Mutual Learning for Underwater Salient Object Detection. IEEE Trans. Image Process..

[57] Zhou W., Tang B., Dong X., Qiang F. (2026). Prompt Then Refine: Prompt-Free SAM-Enhanced Collaborative Learning Network for Detecting Salient Objects in Underwater Images. IEEE Trans. Neural Netw. Learn. Syst..

[58] He K., Fan H., Wu Y., Xie S., Girshick R. Momentum Contrast for Unsupervised Visual Representation Learning. Proceedings of the IEEE/CVF Conference on Computer Vision and Pattern Recognition (CVPR).

[59] Xie E., Ding J., Wang W., Zhan X., Xu H., Sun P., Li Z., Luo P. (2022). DetCo: Unsupervised Contrastive Learning for Object Detection. IEEE Trans. Circuits Syst. Video Technol..

[60] Chen T., Kornblith S., Norouzi M., Hinton G. A Simple Framework for Contrastive Learning of Visual Representations. Proceedings of the International Conference on Machine Learning (ICML).

[61] Li X., Wang W., Hu X., Li J., Tang J., Yang J. Generalized Focal Loss V2: Learning Reliable Localization Quality Estimation for Dense Object Detection. Proceedings of the IEEE/CVF Conference on Computer Vision and Pattern Recognition (CVPR).

[62] van den Oord A., Li Y., Vinyals O. (2018). Representation Learning with Contrastive Predictive Coding. arXiv.

[63] Zhou W., Dong L., Ye O., She X., Duan X., Peng Z., Wang S., Zhao N., Guo X. (2024). Coal Mine Underground Drilling Site Object Detection Dataset. Science Data Bank. https://www.scidb.cn/en/detail?dataSetId=93f9246fd5794fc0be7b120a348eccc5.

[64] Lin T.Y., Maire M., Belongie S., Hays J., Perona P., Ramanan D., Dollár P., Zitnick C.L. Microsoft COCO: Common Objects in Context. Proceedings of the European Conference on Computer Vision (ECCV).

[65] Loshchilov I., Hutter F. Decoupled Weight Decay Regularization. Proceedings of the International Conference on Learning Representations (ICLR).

